# Gender Effect in Experimental Models of Human Medulloblastoma: Does the Estrogen Receptor β Signaling Play a Role?

**DOI:** 10.1371/journal.pone.0101623

**Published:** 2014-07-07

**Authors:** Alessandra Ciucci, Daniela Meco, Ilaria De Stefano, Daniele Travaglia, Gian Franco Zannoni, Giovanni Scambia, Riccardo Riccardi, Anna Saran, Mariateresa Mancuso, Daniela Gallo

**Affiliations:** 1 Department of Obstetrics and Gynecology, Catholic University of the Sacred Heart, Rome, Italy; 2 Division of Pediatric Oncology, Catholic University of the Sacred Heart, Rome, Italy; 3 Department of Radiation Physics, Università degli Studi Guglielmo Marconi, Rome, Italy; 4 Department of Histopathology, Catholic University of the Sacred Heart, Rome, Italy; 5 Laboratory of Radiation Biology and Biomedicine, Agenzia Nazionale per le Nuove Tecnologie, l'Energia e lo Sviluppo Economico Sostenibile (ENEA), CR-Casaccia, Rome, Italy; University of Navarra, Spain

## Abstract

**Background:**

The male-to-female sex ratio for medulloblastoma (MB) is approximately 1.5∶1, female gender being also a favorable prognostic factor. This study aimed at evaluating the impact of gender on MB tumorigenesis.

**Methods:**

*In vitro* activity of 17β-estradiol (E_2_), DPN [2,3-bis(4-hydroxyphenyl)-propionitrile, a selective estrogen receptor β (ERβ)-agonist], PPT [4,4′,4″-(4-Propyl-[1H]-pyrazole-1,3,5-triyl) trisphenol, a selective ERα-agonist] or DHT (5 alpha-dihydrotestosterone) was evaluated in three human MB cell lines. D283 Med cells were transplanted into athymic mice.

**Results:**

A significant expression of ERβ, with little or no ERα, and low AR (androgen receptor) was found in MB cell lines. The compounds tested did not affect cell proliferation. In vivo, we observed a significantly lower growth of D283 Med in nude female mice compared to males. At microscopic examination, tumors from females showed a shift towards differentiation, as evaluated by lower nestin, and higher NSE (neuron-specific enolase) and GFAP (glial fibrillary acidic protein) expression compared to males. Tumors from females also showed lower Ki67 and p53 expression. The wild-type ERβ protein (ERβ1) was lost in male tumors, while it was a permanent feature in females, and a strong negative correlation was found between Ki67 and ERβ1 expression. Conversely, tumor levels of ERβ2 and ERβ5 did not significantly differ between genders. Increased levels of cyclin-dependent kinase inhibitor p21 were observed in females, suggesting that estrogen may decrease tumor growth through blocking cell cycle progression. An inhibition of the insulin-like growth factor I (IGF-I) signaling was also evident in females.

**Conclusion:**

We provides mechanistic evidence supporting the idea that ERβ1 signaling may have pro-differentiation and tumor suppressive function in medulloblastomas.

## Introduction

Once thought to be irrelevant regarding steroid hormones action, the cerebellum is now recognized to be affected by these hormones, with the majority of studies focusing on the role of estrogen. Indeed, both ERα and ERβ are expressed in the cerebellum and their expression varies with age [1]. ERα expression is significantly higher in the cerebellum of neonatal rat compared to adults. During development, ERα is mainly confined to Purkinje cells, with peak intensities of immunostaining coinciding with dendritic growth and synapse formation; in the adult, the protein is localized primarily to granule cells, albeit at low concentrations. Similar to ERα, ERβ expression is modulated during cerebellar development. Differentiating granule cells express detectable levels of ERβ throughout development. In Purkinje cells, ERβ reaches a peak during the initiation of axonal and dendritic growth, slightly decreasing over time to the level maintained throughout adulthood [2]. Overall, these findings indicate that in developing cerebellar neurons and glia, ERβ expression increases after completion of mitosis, is expressed at highest levels during periods of differentiation characterized by migration or extension of processes, and is then decreased to lower levels in mature cells. In the adult, high levels of ERβ are expressed in Purkinje cells and in the granule cell layer [2]. On the other hand, little is known on the specific role of androgens in cerebellum, with only limited data suggesting that androgens may also have a role in cerebellar development [3]. In this context, it is noteworthy that Purkinje cells express various hormone synthesizing enzymes, including the key enzyme in estradiol formation, cytochrome P450 aromatase, implying that estrogens are also synthesized locally from androgens in the brain [1]. Largely, there is growing evidence that estrogen contributes to sex differences in various cerebellar diseases, including cancer, although whether this is due to sex differences in cerebellar physiology and/or differences in hormone production between males and females remains to be fully determined.

Medulloblastoma (MB), a highly malignant primitive neuroectodermal tumor of the cerebellum, represents about 20% of all childhood primary CNS tumors. The peak age at presentation is 3–6 years, with only 25% of patients between 15 and 44 years of age. In adults, the disease is much less common, comprising only 1% of primary brain tumors [4], [5]. Epidemiological studies have shown that male sex is a risk factor for MB, irrespective of age, race or region of the world [6], [7], with approximately 65% of patients being males. Besides this different susceptibility, other authors have found that female gender is also a significant favorable prognostic factor in MB, with girls having a much better outcome [8]–[10]. These observations clearly suggest the existence of sexually dimorphic mechanisms that broadly impact on tumorigenesis and tumor progression.

In line with these epidemiological findings, using a well characterized mouse model of radiation-induced MB (the Patched1 heterozygous mice, *Ptch1^+/−^*), we previously showed a protective action of estrogen during early stages of MB development; indeed, susceptibility to MB development was significantly increased in OVX *Ptch1^+/−^* females, and restored to levels observed in control mice after estrogen replacement [11]. We next investigated the molecular mechanisms by which estrogen might influence tumor progression, and showed that ERβ, but not ERα, is involved in modulation of MB development by estrogens [12]. These effects were achieved via activation of anti-proliferative and pro-apototic pathways.

The present study aimed at evaluating the impact of gender on preclinical models of human MB. Here, a significant expression of the ERβ, with little or no ERα, and low androgen receptor (AR) was found in three human MB cell lines (D283 Med, Daoy, and UW228). Cell proliferation was not affected by 17β-estradiol (E2), DPN [2,3-bis(4-hydroxyphenyl)-propionitrile, a selective ERβ-agonist], PPT [4,4′,4″-(4-Propyl-[1H]-pyrazole-1,3,5-triyl) trisphenol, a selective ERα-agonist] or DHT (5 alpha-dihydrotestosterone). *In vivo*, we observed a significantly lower growth of D283 Med in nude female mice compared to males, these latter also showing a shift towards tumor de-differentiation. Results from our study supports the idea that ERβ signaling may play a role in the regulation of MB development and growth.

## Materials and Methods

### Cell culture

The medulloblastoma cell lines D283 Med (derived from a 6-year-old boy) and Daoy (derived from a 4-year-old boy) were purchased from the European Collection of Cell Cultures (ECACC, Salisbury, UK). UW228 cell line (derived from a 9-year-old female) [13] was a generous gift of Dr. Karel Zitterbart. Daoy were cultured in RPMI 1640 medium (Lonza, Basel, Switzerland), D283 Med and UW228 were grown in Dulbecco's modified Eagle's medium/F12 (Lonza). The medium was supplemented with 10% fetal bovine serum (FBS, Lonza), 2 mM glutamine and antibiotics (100 mg/ml streptomycin and 100 IU/ml penicillin) (Lonza). All cultures were maintained at 37°C under a humidified atmosphere of 5% CO2 and 95% air.

### Proliferation assay

D283 Med (1.0×10^5^ per well), Daoy (8.0×10^4^ per well), and UW228 (8.0×10^4^ per well) cells were seeded in 6-well plates in complete culture medium. Cells from cultures in log phase of growth were used. After overnight incubation, the medium was changed to phenol-red free medium supplemented with 5% CS-FBS (charcoal-stripped serum) and containing various concentrations of 17β-estradiol (E_2_, Sigma-Aldrich, St. Louis, MO), 2,3-bis(4-hydroxyphenyl)-propionitrile (DPN, Tocris Bioscience, Ellisville, MO), 4,4′,4″-(4-Propyl-[1H]-pyrazole-1,3,5-triyl) trisphenol (PPT, Tocris Bioscience) or 5 alpha-dihydrotestosterone (DHT, Sigma-Aldrich). The substances were dissolved in absolute ethanol and diluted in the appropriate culture medium immediately before use. Control cells received the same amount of diluent. The medium was renewed after 48 hours. At 96 hours of incubation, cells were harvested by trypsinisation, and viable cells were counted using Nucleocounter (Chemometec, Allerod, Denmark). All experiments were performed at least three times in duplicate. To validate our experimental conditions, the proliferation of MCF-7 cells (ECACC) was assessed following treatment with E_2_, DPN, PPT and DHT. Also, the proliferation of Daoy cells after GANT58 (a specific Gli1 inhibitor, Calbiochem, San Diego, CA) and IGF-1 (Calbiochem) treatments was evaluated to confirm that the growth of MB cells can be modulated in our culture conditions. Substances were dissolved according to manufacturer's instructions.

### Mice

Male (n = 12) and female (n = 12) athymic mice [Athymic Nude-nu], 6 weeks old, were obtained from Charles River S.r.l. (Lecco, Italy), and housed under controlled conditions. The UKCCCR guidelines [14] for the welfare of animals in experimental neoplasia were followed. The study was approved by the Animal Care and Use Committee of the Università Cattolica del Sacro Cuore (Rome, Italy, Prot. Pdc. CESA/A/26/2012), and by the Italian Ministry of Health.

### MB induction

At eight weeks of age all mice received 0.1 ml subcutaneous injection of 3×10^7^ D283 Med cells in phenol red-free/growth factor-reduced Matrigel (BD Biosciences, San Diego, CA). Inoculated animals were observed daily with tumors measured at least twice per week using vernier calipers. Tumor weight was calculated from two dimensional measurements (mm) [15], [16]: Tumor weight  =  length × width^2^/2. Upon health decline (i.e., severe weight loss, paralysis, ruffling of fur, inactivity), they were euthanized to minimize suffering. All remaining animals were sacrificed 7 weeks after tumor inoculation (i.e. day 49 of the study). At necropsy, all tumors were removed, weighed and fixed in 4% buffered formalin for histology, or stored at −80°C for immunoblot analysis.

### Western Blot Analysis

Tumor tissues were homogenized in cold RIPA lysis buffer containing protease plus phosphatase inhibitors. Samples were incubated on ice for 30vmin, centrifuged, and the supernatant protein quantified by the Bradford assay (Biorad Laboratories Inc.). Equal amounts of protein (40 µg/sample) were separated by SDS polyacrylamide gel (Bio-Rad Laboratories, Hercules, CA), and transferred onto PVDF membranes (Immobilon-P transfer membrane, Millipore, Billerica, MA). The membranes were blocked for 1 hour with 5% (w/v) nonfat dry milk in Tris Buffered Saline containing 0.1% Tween 20 (TBST) and incubated with primary antibodies: ERα (clone 6F11, Santa Cruz, CA, USA); ERβ (clone H-150, Santa Cruz, directed against the N terminus of ERβ and thus referring to total protein level, including both full-length ERβ1 and truncated isoforms); AR (Biorbyt, Cambridge, UK); p-21 (187, Santa Cruz); cyclin D1 (A12, Santa Cruz); Cyclin E (M20, Santa Cruz); anti-phospho-p44/42 mitogen-activated protein kinase (p-p44/42 MAPK) (Thr202/Tyr204) (Cell Signaling Technology, Boston, MA, USA), and anti-total-p44/42 MAP kinase (Cell Signaling Technology); β-actin (A5441, Sigma-Aldrich), in TBST with 5% albumin bovine serum, at 4°C overnight. After washing three times with TBST, the membranes were labeled with horseradish peroxidase-conjugated secondary antibodies for 1 hour at room temperature. Specific proteins were visualized with the ECL Western blotting system according to the manufacturer's instructions (Pierce Biotechnology, Rockford, IL). β-actin Western blot analysis was performed as a control of equal sample loading.

### Immunohistochemistry

Immunohistochemical analysis was carried out on 3-µm thick paraffin sections as described [17]. Antibodies used include: anti-ERα (clone 6F11, Santa Cruz, dilution 1∶100); anti-ERβ1 (clone PPG5/10; Dako, Glostrup, Denmark, dilution 1∶50); anti-ERβ2 (clone 57/3, Serotec Ltd, Oxford, United Kingdom, dilution 1∶100), and ERβ5 (clone 5/25 Serotec, dilution 1∶100); anti-AR (clone AR441, Abcam, Cambridge, UK, dilution 1∶50); anti-Ki67 (clone MIB-1, M7240, Dako, dilution 1∶50); anti-p53 (clone DO-7, Dako, dilution 1∶50); anti-nestin (AB5968, Abcam, dilution 1∶300); anti-neuron specific enolase (NSE, clone BBS/NC/VI-H14, Dako, ready to use); anti-glial fibrillary acidic protein (GFAP, Z0334, Dako, dilution 1∶500); anti-phospho-IGF-I Receptor b (Tyr1131) (polyclonal, Cell Signaling Technology, dilution 1∶50).

We utilized human healthy liver to confirm specificity of the ER primary antibodies used, showing that in this tissue, ERβ1 and ERβ2 isoforms are not expressed, while specific nuclear ERβ5 immunoreactivity is detected. This pattern of ERβ isoforms immunoreactivity is consistent with previous descriptions [18]. Representative pictures are shown in Figure S1. The specificity of the antibodies directed against the ERβ isoforms has been also validated by other Authors using Western blotting, and these antibodies have been widely used in clinical studies by our and other groups as well [19]–[27]. Staining without primary antibody was used to validate the specificity of the secondary antiserum. Immunohistochemical scoring was carried out by investigators blinded to treatment groups. The number of positive (brown stained) cells was determined as the percentage of the total number of cells counted in 5 separate fields of 100 cells, in non-necrotic areas of 6 tumors, in each group.

### Evaluation of immunohistochemical data

Scoring of ERβ1, ERβ2 and ERβ5 was evaluated as previously reported [26]–[28]. Briefly, the mean percentage of stained cells was categorized as follows: 0 = negative, 1 = 1–10%, 2 = 11–33%, 3 = 34–66%, 4 = 67–100%. The intensity of staining was also evaluated and graded from 1–3, where 1 = weak staining, 2 = moderate staining, and 3 = strong staining. The two values obtained were multiplied to calculate a receptor score (maximum value 12). Positivity for Ki67 and p53 was expressed as the percentage of cells with positive nuclear staining. Immunoreactivities for nestin, enolase, GFAP and p-IGF-IR, were scored as follows. A proportion score (PS) was defined as the percentage of positively stained tumor cells: 0 = negative; 1 = <30%, 2 = 30–60%, 3 = >60%. An intensity score (IS) was defined as the staining intensity of positive tumor cells: 1 = weak staining; 2 = moderate staining; 3 = strong staining. The immunohistochemical (IHC) score was determined as the product of the PS and IS [29].

### Statistics

Tumor growth data were analyzed by repeated-measures ANOVA, followed by the Bonferroni method as post-test. The remaining data were analyzed for homogeneity of variance using an F test. If the variances were heterogeneous, log or reciprocal transformations were made in an attempt to stabilize the variances. If the variances remained heterogeneous, a non-parametric test such as the Mann–Whitney U test was used. Correlations between variables were identified employing the Spearman's rank correlation. P values are for two-sided tests; *p* values ≤0.05 were considered statistically significant. Analyses were performed using GraphPad Prism version 5.0 for Windows (GraphPad Software, San Diego, CA).

## Results

### *In vitro*

**Figure 1:**
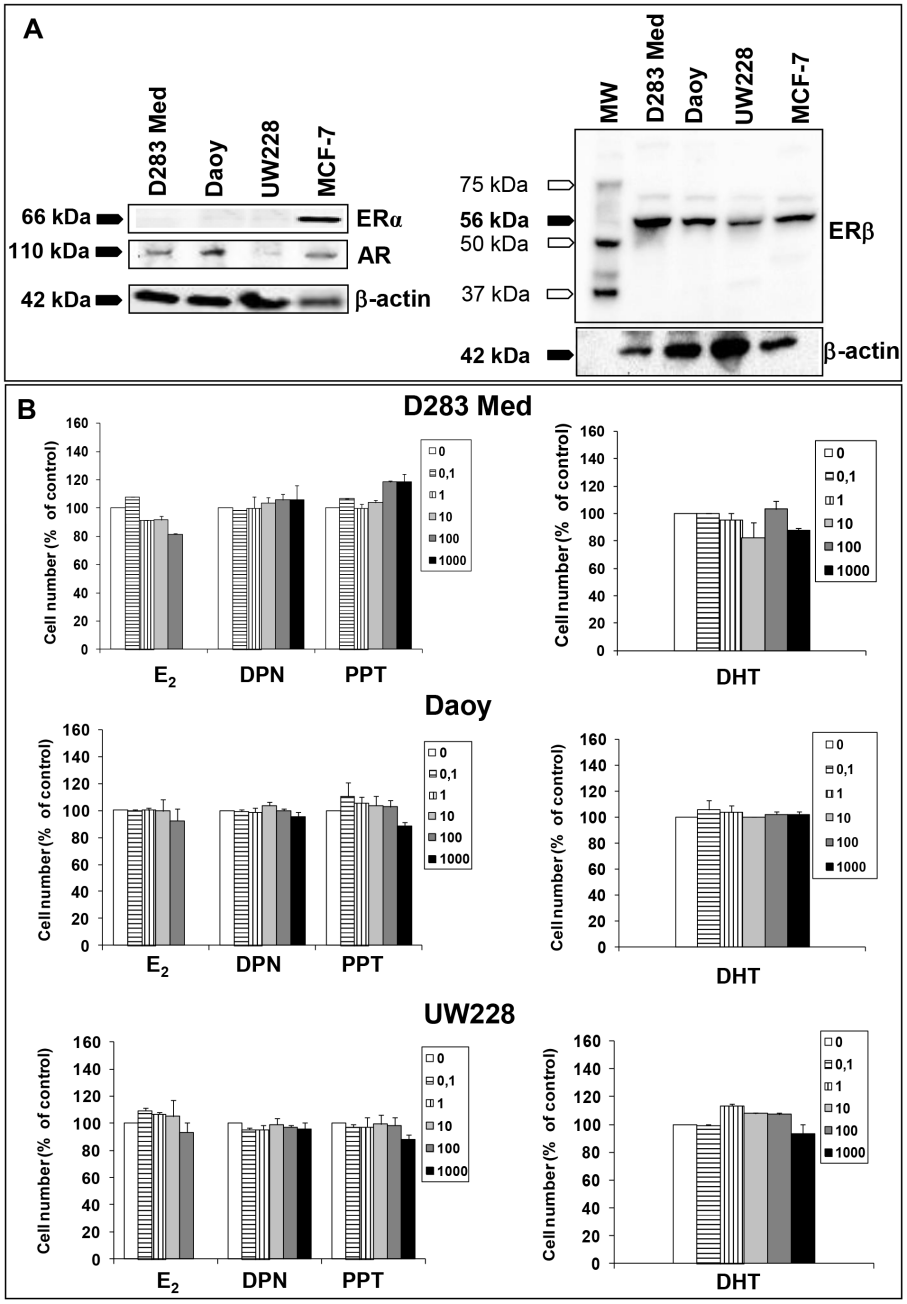
*In vitro* experimental studies. **A)** Comparative Western blot analysis of ERs and AR expression in MB cells. Shown are representative Western blots of SDS-PAGE gels containing whole-cell protein lysates from D283 Med, Daoy and UW228 cells. ERα was undetectable in these MB cell types. A major ERβ immunoreactive species of 56 kDa was present in all MB cells. The AR was expressed in D283 Med and Daoy, but not in the female-derived MB cell line UW228. MCF-7 cells were used as positive control. MW, molecular weight. **B)** Effects of E_2_, the ERβ-selective agonist DPN, the ERα-selective agonist PPT and DHT (5 alpha-dihydrotestosterone) on the growth of MB cell lines. Cells were seeded in phenol-red free medium supplemented with 5% charcoal stripped FCS, containing various concentrations of substances. Concentrations are expressed in nanomolar. Control cells received the same amount of diluent. The medium was renewed after 48 hours. At 96 hours of incubation viable cells were counted using Nucleocounter. All results are expressed as the mean ± SEM derived from at least three different experiments.

For each MB cell culture model, immunoblot analyses were used to evaluate the expression of ERα, ERβ and AR. While ERα was undetectable, total ERβ protein expression was identified in each cell line. A low AR expression was found in D283 Med and Daoy, but not in the female-derived MB cell line UW228 (Figure 1A).

Proliferation of D283 Med, Daoy, and UW228 cells was evaluated 96 hours following the treatment with E_2_, DPN, PPT or DHT (Figure 1B). Results obtained showed that E_2_ did not induce MB cell proliferation at any concentration tested. The lack of effect was also confirmed following longer exposure (7 days, data not shown). In addition, both the ERβ- and the ERα-selective agonists used (DPN and PPT, respectively) did not influence viable cell numbers, up to 1000 nM (Figure 1B). Finally, DHT, the physiologically active form of testosterone, did not modulate human MB cell lines proliferation. MCF-7 cells used as positive control gave results consistent with literature data (Figure S2) [30]–[35]. GANT58 (a specific Gli1 inhibitor, negative control) and IGF-1 (positive control) were also used to confirm that the growth of MB cells can be modulated under our experimental conditions (Figure S3).

### *In vivo* - Effect of gender on MB growth

**Figure 2:**
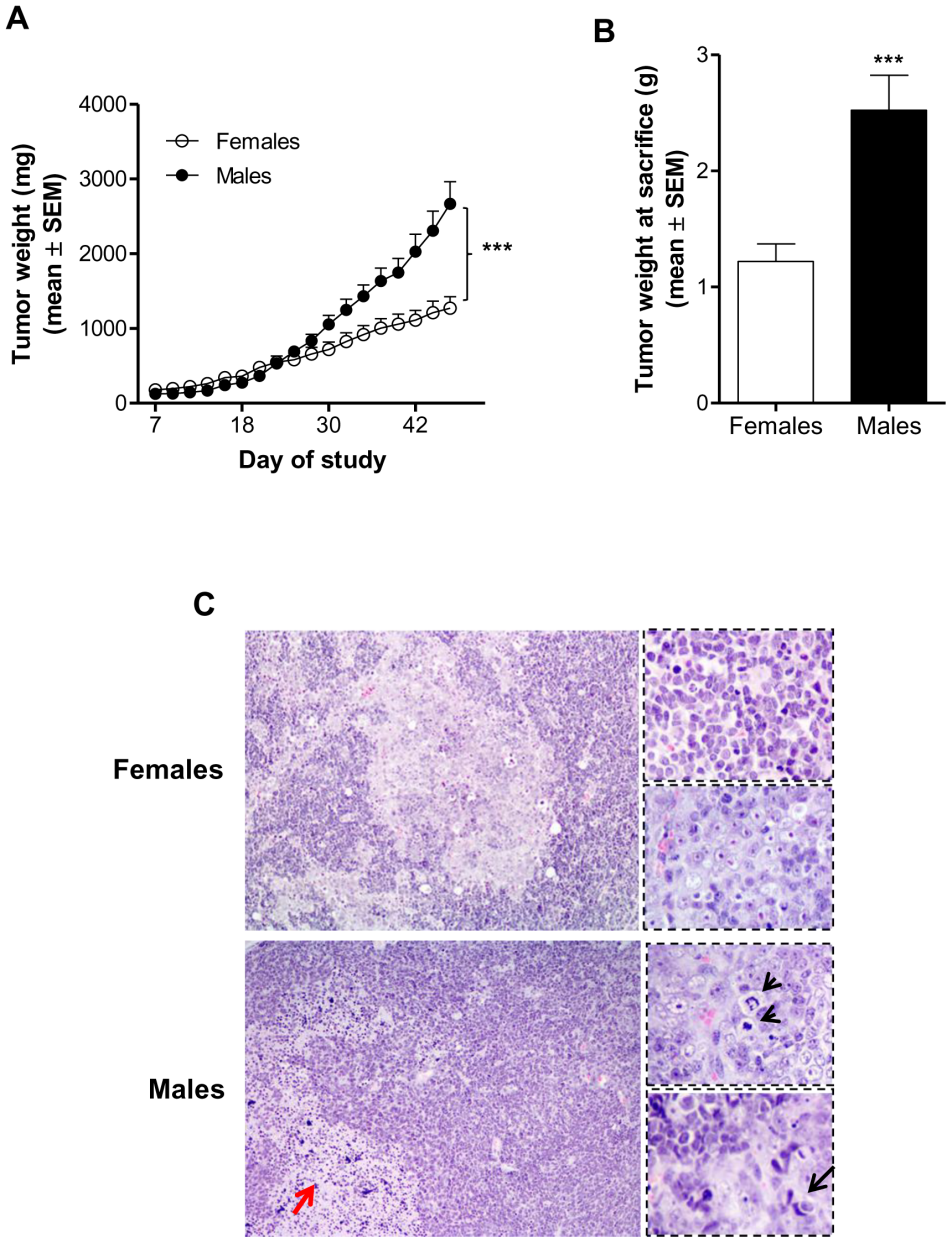
Effect of gender on *in vivo* growth of D283 Med. **A)** D283 Med tumor growth during and **B)** at the end of the study in the experimental groups. Tumors in females were significantly smaller compared to males, starting from day 32 of the study (n = 12/group). All results are expressed as the mean ± SEM. ***p<0.001. **C)** Representative H&E stained section at different magnification of tumors from females and males (20x, 40x). Tumors in females were almost desmoplastic, distinctive for having a biphasic architecture with nodular arrangements of tumor cells. On the other hand, anaplastic lesions characterized by conspicuous areas of necrosis (red arrow), high mitotic activity (arrow heads) and nuclear molding (black arrow) were typical features in male mice.

To assess the role of gender on MB growth, we xenotransplanted intact females and males with a suspension of D283 Med, and measured tumor dimensions up to seven weeks after tumor inoculation. Repeated-measures ANOVA, followed by Bonferroni's test showed that tumors in females were significantly smaller compared to males, starting from day 32 of the study (Figure 2A and 2B). At microscopic examination, a marked difference in tumor morphology was observed between females and males (Figure 2C). Indeed, while tumors in females were almost desmoplastic, characterized by a biphasic architecture with nodular arrangements of tumor cells, anaplastic lesions characterized by cells with large, pleomorphic nuclei with prominent nucleoli, nuclear molding, high mitotic activity, and conspicuous areas of necrosis were typical features in male mice.

### *In vivo* – Hormone receptor profile

**Figure 3:**
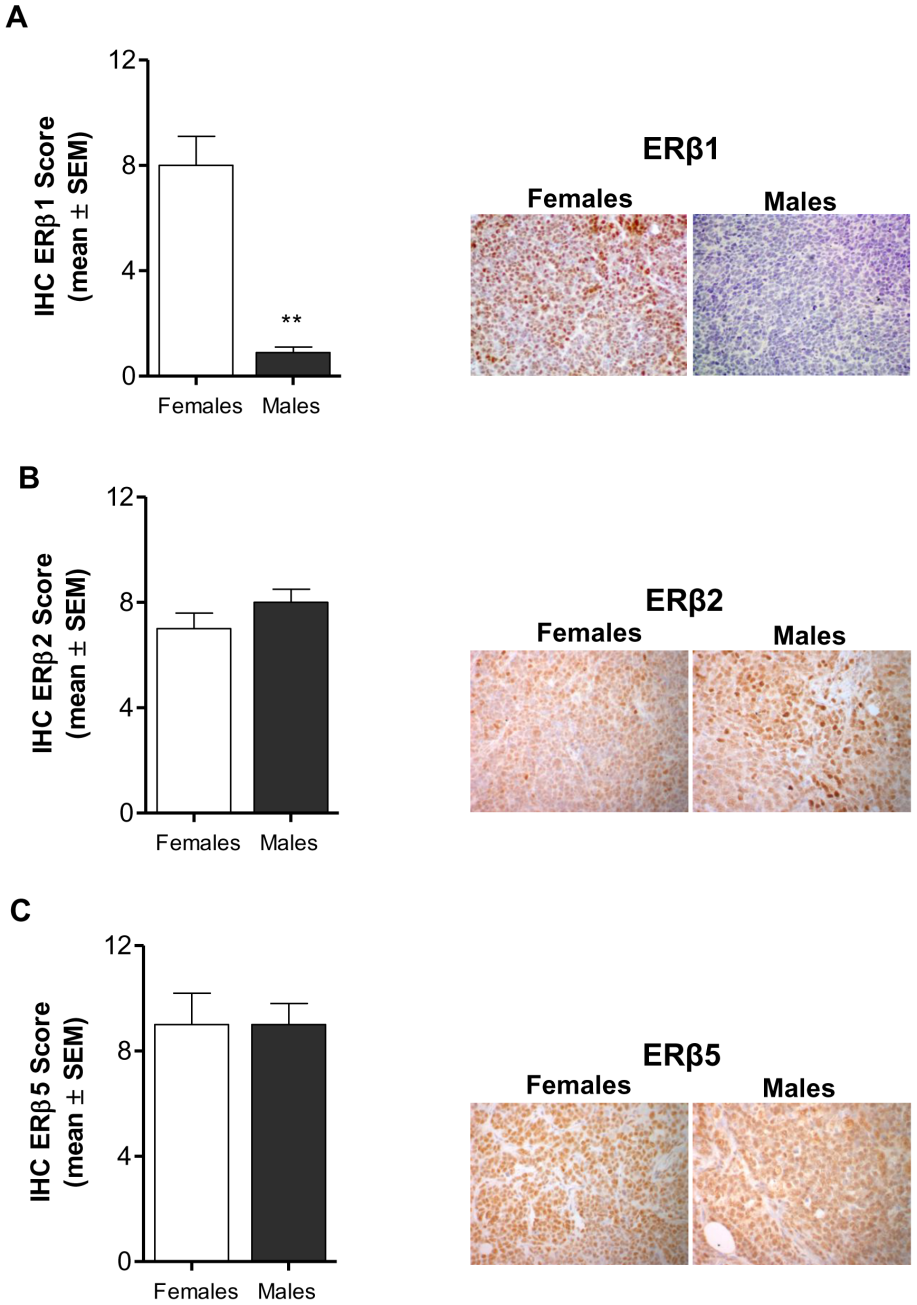
Effect of gender on ERβ1, ERβ2 and ERβ5 expression in D283 Med xenografts. **A) **Immunostaining for ERβ1 was significantly higher in tumors from females compared to males (**p<0.01); **B)** and **C)** Tumor levels of ERβ2 and ERβ5 did not significantly differ between females and males. All results are expressed as the mean ± SEM (n = 6 tumors/group). Representative stained section of tumors from females and males are shown (magnification 40x). IHC, immunohistochemical score.

To shed light on the mechanisms for a potential influence of sex on the biology of MB we examined hormone receptor expression profile in tumors from both female and male mice. Specifically, immunohistochemistry was used for evaluation of ERα, the wild-type ERβ (ERβ1), two ERβ splice variant isoforms (ERβ2, ERβ5) [36], and AR expression. No detectable ERα was observed in tumors from intact females and males (data not shown). On the other hand, we found that ERβ1 expression was significantly higher in tumors from females compared to males (p<0.01, Figure 3A), in which very weak expression was detected. The antibody we used in the present study actually recognizes both the ‘long’ and ‘short’ forms of ERβ1 which are formed by translation from different ATGs in the mRNA [37], but, according to Hall and McDonnell [38], the activities of these forms are indistinguishable with respect to response to ER agonists and antagonists. We also observed that tumor levels of ERβ2 and ERβ5 did not significantly differ between genders (Figure 3B and 3C). Finally, we found extremely low levels of AR (<5%) in male tumors, while protein expression lacked in tumors from females (data not shown).

### *In vivo* – Proliferation and apoptosis

**Figure 4:**
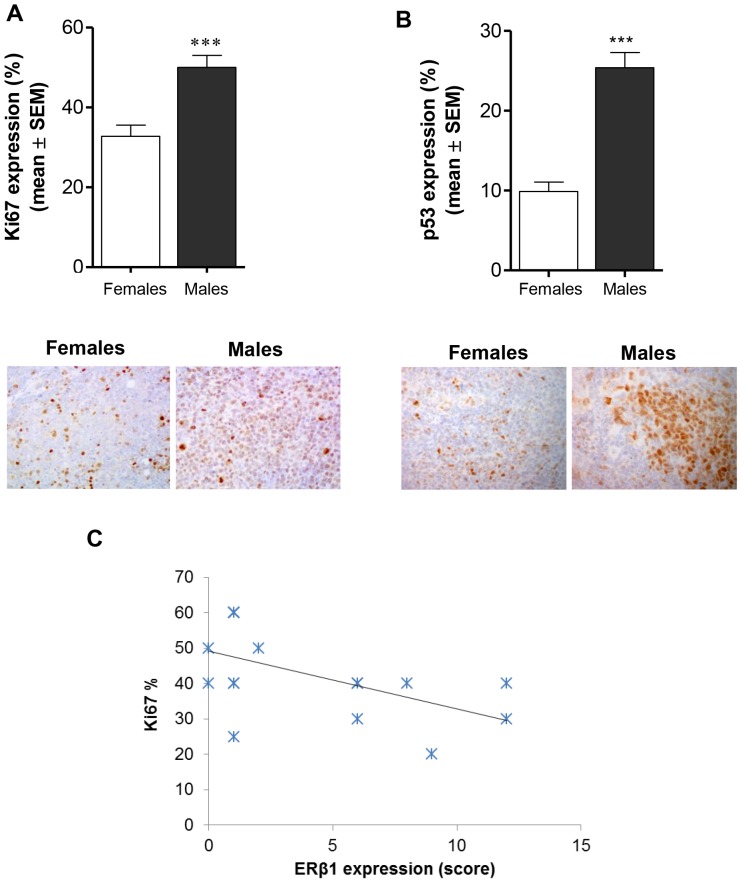
Effect of gender on Ki67 and p53 expression in D283 Med xenografts. **A)** Immunostaining for Ki67 was significantly lower in tumors from females compared to males (***p<0.001); **B)** Immunostaining for p53 was significantly lower in tumors from females compared to males (***p<0.001). All results are expressed as the mean ± SEM (n = 6 tumors/group); representative stained section of tumors from females and males are shown (magnification 40x). **C)** The Spearman rank correlation showed a significant negative correlation between ERβ1 and Ki67 levels (*p<0.05).

To assess whether the lower growth potential of MB lesions in female mice was accompanied by a lower proliferation, we compared the tumor proliferation rate of female and male mice. Immunohistochemistry was carried out on tumor sections using an antibody directed against the proliferation marker Ki67. The mean values of positive cells were 32.8±2.8% and 50.0±3.0% (mean ± SEM) in females and males respectively, showing a significantly lower proliferative potential of lesions from female mice when compared to males (*p*<0.001, Figure 4A). Notably, the Spearman rank correlation showed a significant negative correlation between ERβ1 and Ki67 levels (r = −0,5073 *p*<0.05) (Figure 4C).

Although immunostaining does not allow to distinguish wild-type from mutated p53, expression of p53 is often used as a surrogate marker for alterations in the functional status of p53. Indeed, a recent study using the same antibody as our, showed that, in clinical medulloblastoma samples, high levels of detectable p53 protein may correlate with point mutations [39]. Thus, we examined p53 protein expression in tumors from each experimental group, as indicator of an aggressive phenotype. Notably we found that the mean values of positive cells were significantly lower in females (9.9±1.59) compared to males (25.4±1.9) (mean ± SEM, p<0.001, Figure 4B).

### *In vivo* – Analysis of signal proteins associated with cell cycle regulation and of mitogenic signalling candidates

**Figure 5:**
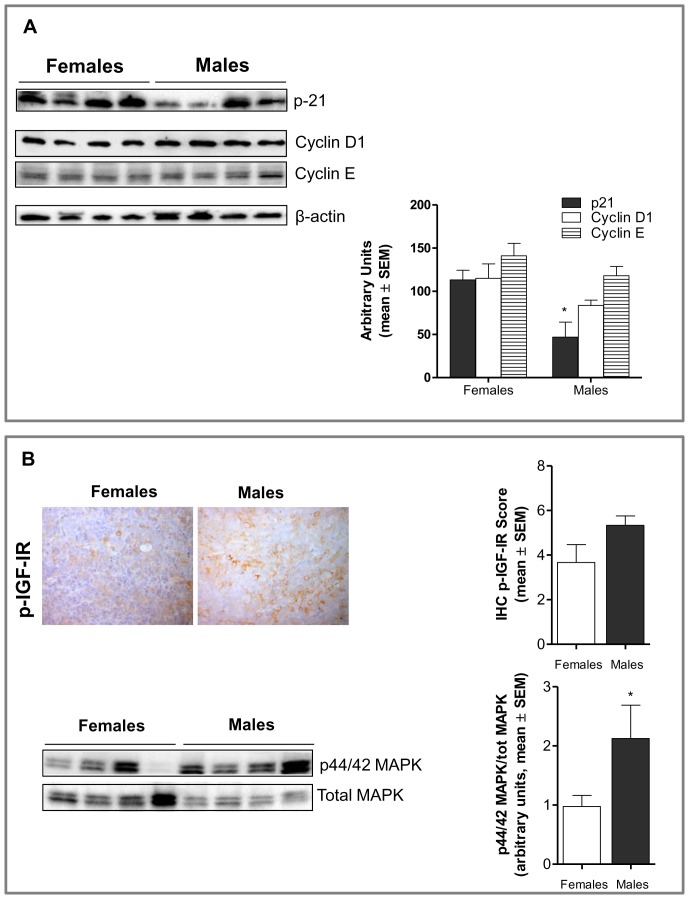
Effects of gender on signal proteins associated with cell cycle regulation and on IGF-IR signaling in D283 Med xenografts. **A)** Densitometric analysis of normalized p21, Cyclin D1 and Cyclin E expression in tumors. p21 protein levels were significantly higher in female tumors as compared to males (*p<0.05), while no differences were observed in Cyclin D1 and Cyclin E expression. All results are expressed as the mean ± SEM (n = 4 tumors/group). **B-upper panel)** Representative pictures showing p-IGF-IR expression in female and male tumors. Magnification 40x.Tumors from females were characterized by lower expression when compared to males. All results are expressed as the mean ± SEM (n = 6 tumors/group). IHC, immunohistochemical score. **B-lower panel)** Densitometric analysis of p44/42 MAPK/total MAPK ratio in tumors. Lower levels of p44/42 MAPK were detected in female tumors as compared to males (*p<0.05). All results are expressed as the mean ± SEM (n = 4 tumors/group).

Using WB analysis, we next investigated the expression of the p53-downstream target, p21 (a cell-cycle arresting factor) [40] in tumor xenografts. Results obtained showed a significantly higher protein expression in female tumors as compared to males (p<0.05, Figure 5A). We went on to investigate the regulation of important protein governing proliferation in the G1-S phase, and no significant differences between genders were found in Cyclin D1 and E levels (Figure 5A).

Since the insulin-like growth factors (IGF) and their cognate receptors (IGFRs) have been shown to play an important role in MB formation and growth [41], we analyzed the expression of phosphorylated IGF-IR, and the potential activation of key signal transduction pathways downstream of IGF-IR, such as the cascade of ERK/MAPK in xenotrasplanted tumors. We found that female tumors showed a lower p-IGF-IR expression compared to males, although this difference only approached statistical significance (IHC, 3.7±0.8 *versus* 5.3±0.4, mean ± SEM, p = 0.09; Figure 5B). In addition, tumors from female mice demonstrated a significantly lower p44/42 MAPK/total MAPK ratio than males (i.e., 0.98±0.18 *versus* 2.13±0.6, mean ± SEM, p<0.05; Figure 5B).

### *In vivo* – Markers of tumor differentiation

**Figure 6:**
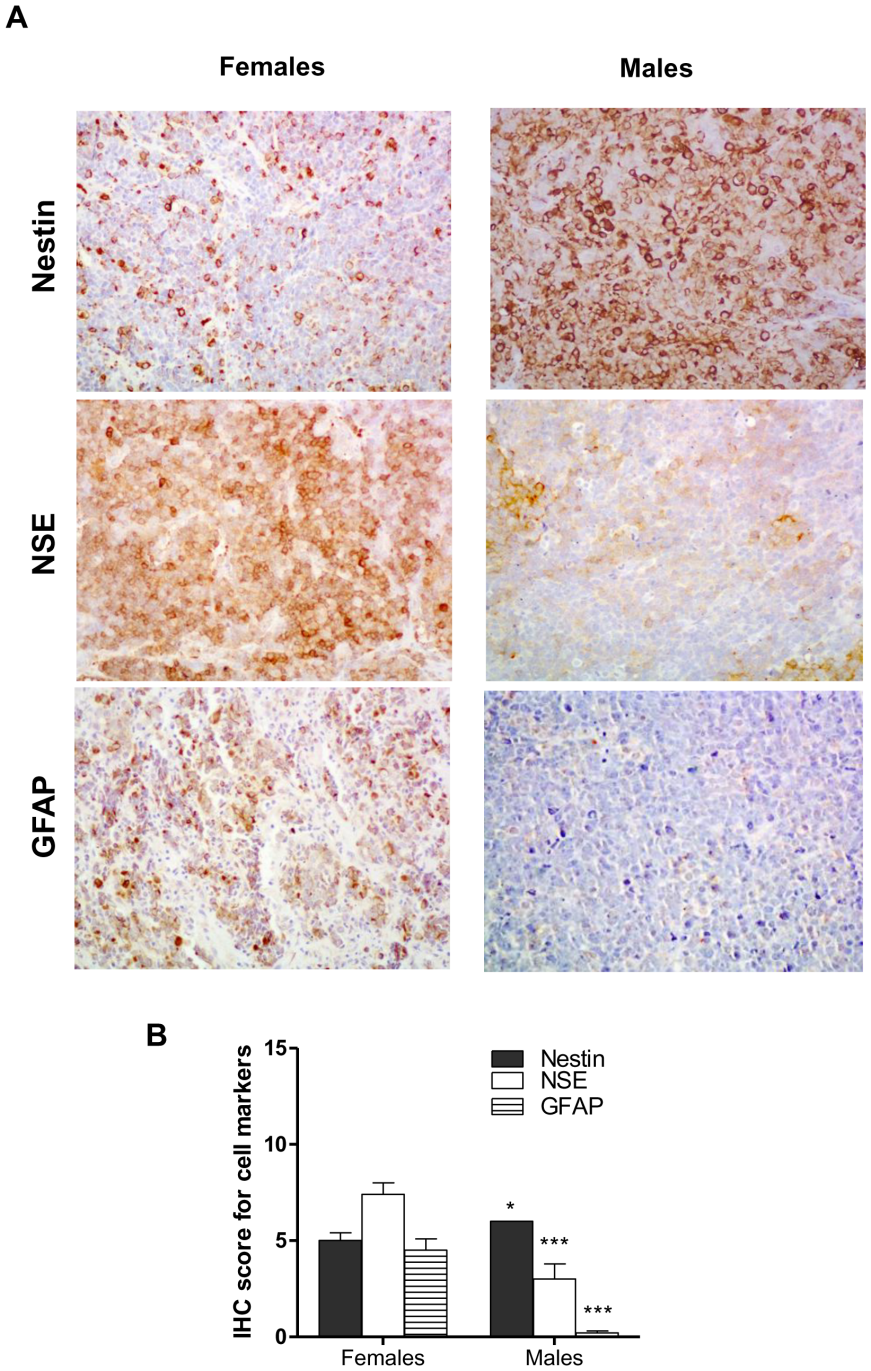
Effects of gender on immunohistochemical markers of tumor differentiation in D283 Med xenografts. **A)** Representative pictures showing tumors expression of nestin, NSE (neuron-specific enolase) and GFAP (glial fibrillary acidic protein) in female and male mice. Magnification 40x. **B)** Tumors from females were characterized by significantly lower (*p<0.05) nestin along with significantly higher NSE and GFAP protein expression (***p<0.001 for both parameters) when compared to males. All results are expressed as the mean ± SEM (n = 6 tumors/group). IHC, immunohistochemical score.

Finally, nestin, NSE and GFAP were used as immunohistochemical markers to compare the degree of tumour differentiation between female and male xenotransplanted mice. Immunohistochemical analysis showed significantly lower nestin levels in tumors from females compared to males (*p*<0.05, Figure 6A and 6B), along with higher NSE and GFAP expression (*p*<0.001, for both parameters, Figure 6A and 6B). Besides, WB analysis also showed a significantly lower vimentin expression in tumors from females when compared to males (data not shown).

## Discussion

Despite the male preponderance of medulloblastoma and reports of female gender as a significant favorable prognostic factor, there has been little investigation into hormonal influences on tumor development. To the best of our knowledge data from experiments described here are the first to support a role of gender in medulloblastoma growth, thus reflecting the clear gender effect in MB susceptibility in both children and adults, and indicating that this body of accumulated evidence, and the conclusions drawn from it, warrant close reevaluation.

Indeed, we found that the growth of D283 Med xenografts was significantly lower in nude female mice when compared to males, tumors from males also exhibiting clear features of anaplasia and a higher proliferative index. Consistent with these data, we observed a more differentiated phenotype, with significantly reduced malignancy, in tumors from females, as shown by a decreased stem cell population (nestin has been identified as a stem like cell marker for medulloblastoma) [42] and a substantial increase of neuronal (NSE) and glial (GFAP) differentiation markers. In this respect, our results are in line with previous findings observed in some human specimens showing that medulloblastomas have a capacity of differentiation along neuronal and glial lines [43]–[45].

To shed light on the mechanisms responsible for a potential influence of sex on the biology of MB we examined hormone receptor expression profile in tumors from both male and female mice. We demonstrated that tumors from both groups were virtually negative for ERα and AR. On the other hand, we found that the expression of ERβ isoforms was differentially regulated in tumors from males and females. Indeed, while ERβ2 and ERβ5 were consistently expressed in all tumors independently of gender, ERβ1 was completely lost in tumors from males, but maintained in females. ERβ1 is the full-length receptor coded by exons 1–8. ERβ2–5 share exons 1–7 with ERβ1, but display unique sequences instead of exon 8. These differences in the C-terminal part of ERβ2–5 determine a truncation of the ligand binding domain and ablation of the ligand-dependent activation function AF-2. Therefore, ERβ1 is thought to be the only fully functional isoform that is able to bind ligand, whereas ERβ2, -β4, and -β5 do not form homodimers and have no innate activities of their own, but may modulate estrogen action when dimerized with ERβ1 [36]. The functions of each isoform in the normal cerebellum are unclear. However in our study, ERβ1 positivity was inversely correlated with Ki67 in tumor samples, a finding also reported by other Authors in different types of cancers [46]–[48], and reinforcing the belief that ERβ1 is anti-proliferative and pro-apoptotic (at least in some tumors). Notably, in keeping with our findings, Urbanska and colleagues [49] recently reported that well differentiated desmoplastic and neuroblastic human medulloblastomas tend to have higher levels of nuclear ERβ when compared to poorly differentiated medulloblastomas.

To obtain a more complete view of the role of ERβ1 (and ERβ2/ERβ5) in MB growth, we wanted to investigate whether their differential expression in males and females was associated with the regulation of protein involved in cell proliferation and/or apoptosis. To this end, we analyzed p53 protein expression using immunohistochemistry and found that tumors from males had a significantly higher p53 labeling index when compared to females, this suggesting p53 dysfunction in male xenografts. Notably, in line with our preclinical results, abnormalities in this cell cycle pathway have been reproducibly associated with anaplasia and poor outcomes in clinical studies [39], [50], [51]. We next investigated by WB analysis, the expression of the p53-downstream target p21 (a cell-cycle arresting factor) in tumors from both sexes, our data showing higher protein expression in females as compared to males. These data may suggest that induction of p21 in female xenografts could be a feature of p53-mediated tumor growth inhibition. It is worthy to note, however, that p21 expression can also be regulated independently of p53 [52], recent reports showing that ERβ is able to produce a p53-independent, ligand-dependent/independent increase of p21 [48], [53]–[56]. Actually, a very recent study in prostate cancer cell lines indicated that this modulation could be attributed to ERβ1, whereas ERβ2 mediated opposite effect [57], this giving further support to our data on the higher expression of p21 in ERβ1-positive female tumors.

We went on to investigate the regulation of important protein governing proliferation in the G1-S phase. We found no differences between genders in Cyclin E and D1 expression. These results are at least partially in agreement with literature data showing that in colon cancer cell lines ERβ did not modulate Cyclin A and E, while inducing Cyclin D1 [48]. Notably, other Authors also found an ERβ1-related increase in Cyclin D1 [57], but even under these conditions, proliferation was inhibited. Altogether, these findings suggest that the decreased cell proliferation observed in female medullablastomas may be at least partially due to the inhibitory control over the cell cycle exerted by p21 (primarily through direct binding to cyclins and cyclin-dependent kinases), this in turn favoring cellular differentiation [40].

We previously suggested that the lower susceptibility to MB development observed in intact and E2-replaced OVX *Ptch1+/−* females compared to OVX, could be the results of a functional interaction between estrogen- and IGF-I-mediated pathways, since we demonstrated an increase in p-IGF-IR in tumors from OVX, in association with induction of the ERK/MAPKs cascade [11]. In line with our previous findings, we showed here that female tumors expressed lower p-IGF-IR, as well as lower p44/42 MAPK levels, compared with males. Notably, among the multiple signaling pathways associated with MB formation and growth [58], the IGF-IR signaling has been shown to play a crucial role, with various components of the IGF system found to be elevated in tumor tissue, cerebrospinal fluid and peripheral blood of pediatric MB patients [59]. In this context, it worthy to mention that the expression of ERs and IGF-IR is cross-regulated in the brain, and, interestingly, this regulation in the cerebellum appears different from other areas of the brain. Specifically, it has been shown that in the cerebellum ERβ may downregulate IGF-IR expression [60]. Overall, these findings suggest that an interplay exists between IGF and ER signaling, a finding also demonstrated in other systems [61].

On the whole, these results support the hypothesis that the molecular mechanisms by which gender affects medulloblastoma may involve the ERβ signaling, and reinforce the growing body of evidence that an imbalanced ERβ expression might play a pivotal role in the development and progression of many tumor types [36]. In this context, it is worth noting that the lack of direct estradiol effect on cell viability and proliferation may be due to the short-term *in vitro* exposure (i.e. 5 to 7 days) or to the fact that estrogen's action may be partly indirect, involving cells of the tumor microenvironment.

In line with our data, Belcher and colleagues [62] observed a significantly more rapid D283 Med xenografts growth in intact males than in females. However, they also found that exogenous estradiol induced a robust stimulation in both cell culture and xenografts, blocked by the antiestrogen drug Faslodex, thus concluding that estrogens have a positive effect on the development of MB, via ERβ-dependent mechanisms (which is opposite to our hypothesis). At present, no data are available that could help explaining this divergence, although a possible interpretation may be in the use of different experimental protocols. Thus, while overall it is clear that multiple factors might influence the ability of ER signaling to regulate MB growth, to the best of our knowledge, there are no other experimental studies addressing this specific issue and, therefore, further research is clearly needed.

Notably, however, a protective role of estrogen has also been underlined for other brain tumors, with very recent studies suggesting a potential role of ERβ protein expression as an independent favorable prognostic factor in gliomas, and the use of ERβ agonist as therapeutic agent in this disease [63]–[67].

In summary, our study has shown for the first time a significant difference in medulloblastoma growth between sex in a preclinical mouse model of the human disease, thus reflecting the greater prevalence of MB in males compared to females, regardless of age, tumor histology and region of the world. We also provide mechanistic evidence supporting the idea that ERβ1 signaling may have pro-differentiation and tumor suppressive function in medulloblastomas, thus suggesting that functional activation of the ERβ pathways may be a potential therapeutic option for MB. However, additional studies are needed to dissect the molecular mechanisms by which estrogens affect tumorigenesis in the brain.

## Supporting Information

Figure S1 **Staining pattern of the ERβ isoforms in human liver.** In order to confirm the specificity of the ERβ antibodies used in the study, we immunostained sections of human healthy liver, showing that ERβ1 and ERβ2 isoforms are not expressed, while specific nuclear ERβ5 immunoreactivity is detected (magnification 20x and 40x). This pattern of ERβ isoforms immunoreactivity is consistent with previous descriptions [18].
(TIF)

Figure S2 **Proliferation studies.** Effects of E_2_, the ERβ-selective agonist DPN, the ERα-selective agonist PPT and DHT (5 alpha-dihydrotestosterone) on the growth of MCF-7 cell line. Cells were seeded in phenol-red free medium supplemented with 5% charcoal stripped FCS, containing various concentrations of substances. Concentrations are expressed in nanomolar. Control cells received the same amount of diluent. The medium was renewed after 48 hours. At 96 hours of incubation viable cells were counted using Nucleocounter. All results are expressed as the mean ± SEM derived from at least three different experiments.
(TIF)

Figure S3 **Proliferation studies.** Effects of GANT58 and IGF-1 on the growth of Daoy cell line. Cells were seeded in phenol-red free medium supplemented with 5% charcoal stripped FCS, containing various concentrations of substances. Concentrations are expressed in micromolar for GANT58 and in ng/ml for IGF-1. Control cells received the same amount of diluent. At 48 hours of incubation viable cells were counted using Nucleocounter. Data are representative of one experiment done in triplicate (mean ± SEM).
(TIF)
