# Supplementary figures and images for: Gender Effect in Experimental Models of Human Medulloblastoma: Does the Estrogen Receptor β Signaling Play a Role?
Source: PLoS One. 2014 Jul 7;9(7):e101623. doi: 10.1371/journal.pone.0101623 (PMC4084991; doi:10.1371/journal.pone.0101623)

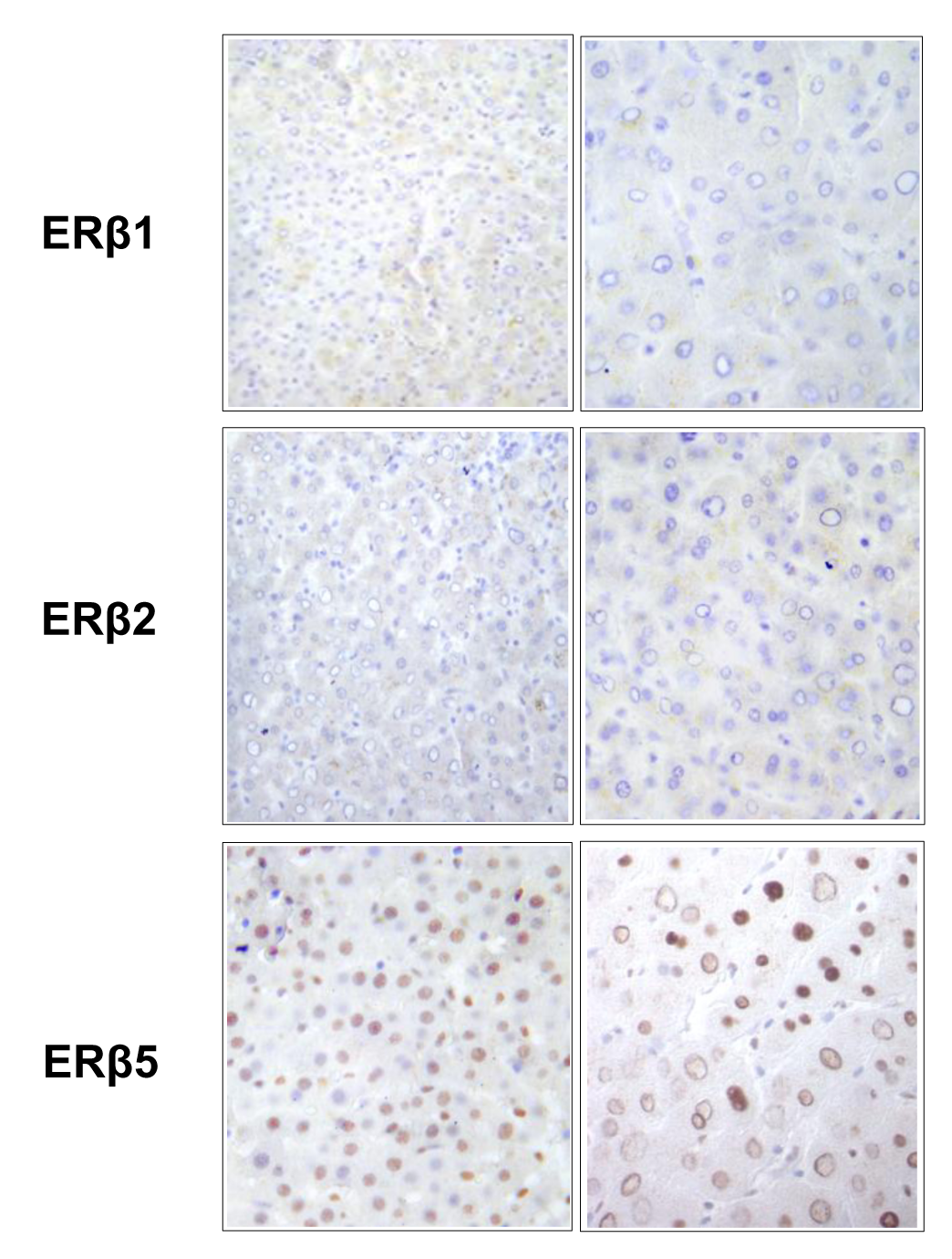

Supplement: Figure S1 — Staining pattern of the ERβ isoforms in human liver. In order to confirm the specificity of the ERβ antibodies used in the study, we immunostained sections of human healthy liver, showing that ERβ1 and ERβ2 isoforms are not expressed, while specific nuclear ERβ5 immunoreactivity is detected (magnification 20x and 40x). This pattern of ERβ isoforms immunoreactivity is consistent with previous descriptions [18]. (TIF) [file pone.0101623.s001.tif]

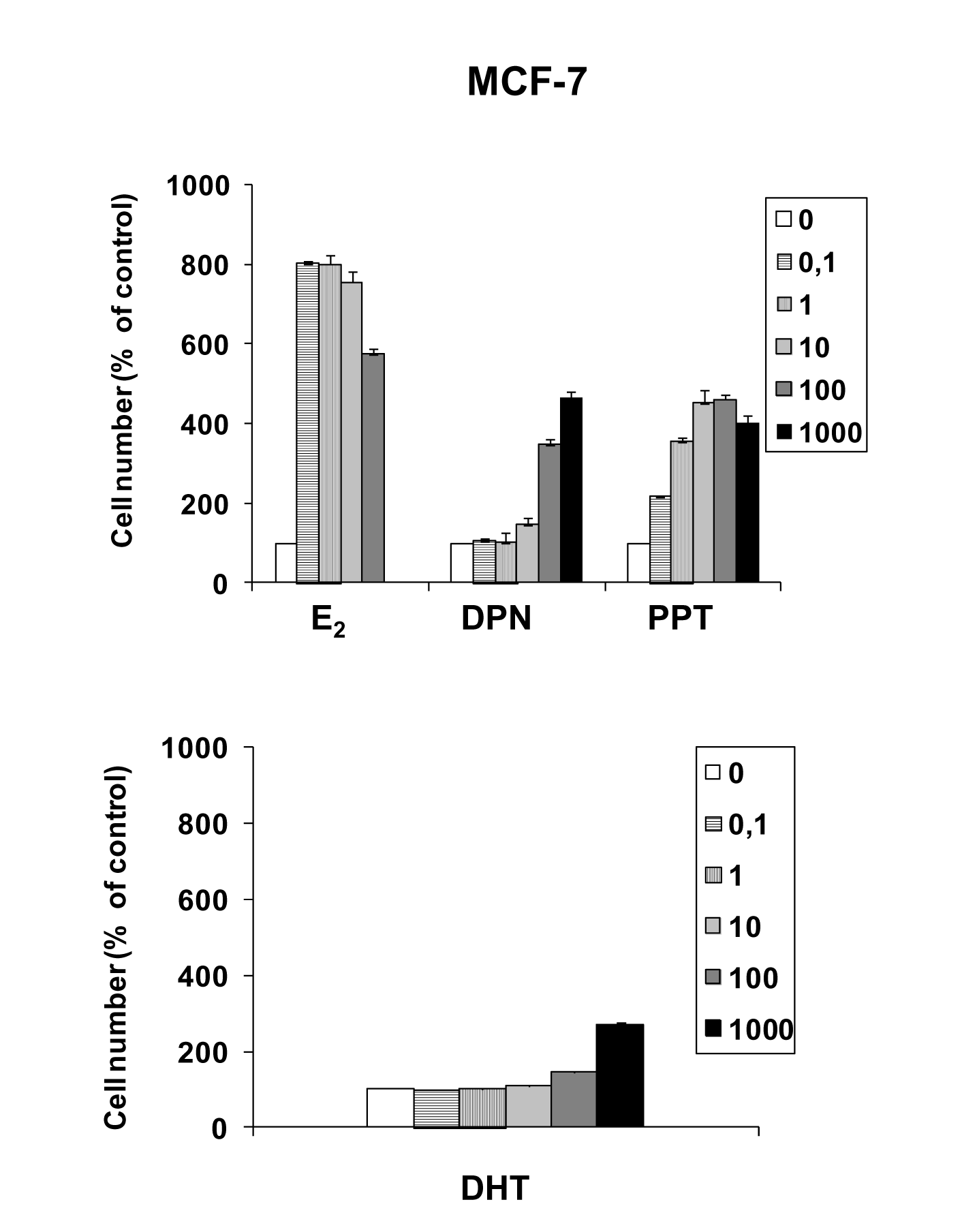

Supplement: Figure S2 — Proliferation studies. Effects of E2, the ERβ-selective agonist DPN, the ERα-selective agonist PPT and DHT (5 alpha-dihydrotestosterone) on the growth of MCF-7 cell line. Cells were seeded in phenol-red free medium supplemented with 5% charcoal stripped FCS, containing various concentrations of substances. Concentrations are expressed in nanomolar. Control cells received the same amount of diluent. The medium was renewed after 48 hours. At 96 hours of incubation viable cells were counted using Nucleocounter. All results are expressed as the mean ± SEM derived from at least three different experiments. (TIF) [file pone.0101623.s002.tif]

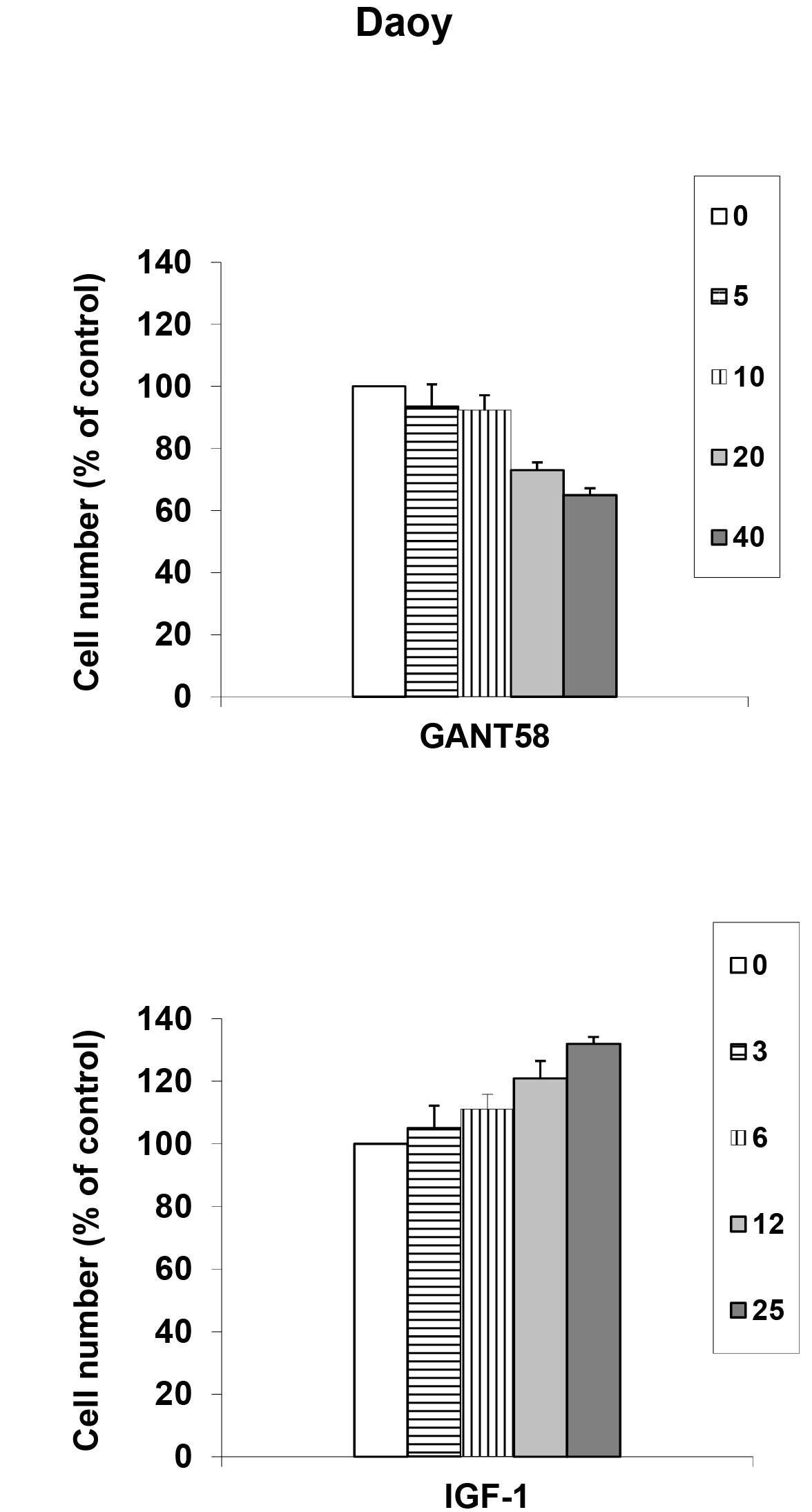

Supplement: Figure S3 — Proliferation studies. Effects of GANT58 and IGF-1 on the growth of Daoy cell line. Cells were seeded in phenol-red free medium supplemented with 5% charcoal stripped FCS, containing various concentrations of substances. Concentrations are expressed in micromolar for GANT58 and in ng/ml for IGF-1. Control cells received the same amount of diluent. At 48 hours of incubation viable cells were counted using Nucleocounter. Data are representative of one experiment done in triplicate (mean ± SEM). (TIF) [file pone.0101623.s003.tif]
